# Blood telomere length gain in people living with HIV switching to dolutegravir plus lamivudine versus continuing triple regimen: a longitudinal, prospective, matched, controlled study

**DOI:** 10.1093/jac/dkad237

**Published:** 2023-08-03

**Authors:** Francesca Lombardi, Alessia Sanfilippo, Massimiliano Fabbiani, Alberto Borghetti, Arturo Ciccullo, Enrica Tamburrini, Simona Di Giambenedetto

**Affiliations:** Fondazione Policlinico Universitario A. Gemelli IRCCS, UOC Malattie Infettive, Largo Agostino Gemelli 8, 00168 Roma, Italia; Dipartimento di Sicurezza e Bioetica, Università Cattolica del Sacro Cuore, Roma, Italia; UOC Malattie Infettive e Tropicali, Azienda Ospedaliero-Universitaria Senese, Siena, Italia; Fondazione Policlinico Universitario A. Gemelli IRCCS, UOC Malattie Infettive, Largo Agostino Gemelli 8, 00168 Roma, Italia; Unità di Malattie infettive, Ospedale San Salvatore, L'Aquila, Italia; Fondazione Policlinico Universitario A. Gemelli IRCCS, UOC Malattie Infettive, Largo Agostino Gemelli 8, 00168 Roma, Italia; Dipartimento di Sicurezza e Bioetica, Università Cattolica del Sacro Cuore, Roma, Italia; Fondazione Policlinico Universitario A. Gemelli IRCCS, UOC Malattie Infettive, Largo Agostino Gemelli 8, 00168 Roma, Italia; Dipartimento di Sicurezza e Bioetica, Università Cattolica del Sacro Cuore, Roma, Italia

## Abstract

**Background:**

Blood telomere length (BTL) is a validated biomarker of aging. ART reduces immunosenescence and has benefits in terms of BTL in people living with HIV (PLWH). However, it has also been observed that ART containing NRTIs, such as tenofovir or abacavir, which are potent inhibitors of human telomerase activity *in vitro*, might negatively affect BTL. Here we investigated the effects on BTL 1 year after switching to a dual therapy (DT) with dolutegravir + lamivudine versus maintaining a standard triple therapy (TT) with a two-NRTI backbone and an anchor drug.

**Methods:**

This was a longitudinal, prospective, matched, controlled study that included virologically suppressed adults on stable three-drug ART who either switched at baseline (BL) to DT or maintained TT. The DT and TT groups were 1:1 matched for age, sex, years since HIV diagnosis, years on ART and anchor drug. BTL was assessed by a monochrome multiplex qPCR at BL and after 48 weeks (W48).

**Results:**

We enrolled 120 PLWH, i.e. 60 participants in each group. At BL, the BTL means were comparable between the two groups (*P* = 0.973). At W48, viro-immunological status was stable and an overall increase in the mean BTL was observed, i.e., +0.161 (95%CI, 0.054–0.268) (*P* = 0.004). However, the within-group analysis showed a significant mean BTL gain in the DT group (*P* = 0.003) but not in the TT group (*P* = 0.656).

**Conclusions:**

In this setting of virologically suppressed PLWH, simplifying to dolutegravir + lamivudine was associated with a higher gain in BTL than maintaining triple therapy after the 1 year follow-up. These findings suggest that as a simplification strategy dolutegravir + lamivudine might have a positive effect on BTL.

## Introduction

Telomere length (TL) is an indicator of cellular senescence and is considered an effective and validated biomarker of aging. It is also a predictor of morbidity and mortality in the general population.^1^

People living with HIV (PLWH) have shorter telomere length (TL) than their HIV-negative counterparts.^2,3^ This TL attrition could be responsible for accelerated aging and an increased risk of age-related non-AIDS morbidity and mortality.^4^

It has been demonstrated that a large proportion of TL shortening occurs early during HIV infection,^5,6^ and TL continues to decline significantly during untreated chronic HIV infection.^7^

Mechanisms that contribute to TL shortening in PLWH can include uncontrolled viral replication and HIV-related sustained immune activation, oxidative stress, inflammation, immunosenescence and the inhibition of telomerase by HIV proteins.^8–10^

Through its initial control of HIV replication, ART partially reverses HIV-associated immunosenescence, which results in an increase in less-differentiated T cells that translates into an increase of mean TL in whole blood, PBMCs and CD8+ T cells.^11–13^ Conversely, unlike an early ART start, delayed ART is associated with significant and sustained blood telomere length (BTL) shortening.^14^

It has also been reported that PLWH successfully treated with long-term ART continue to experience mean BTL gains, suggesting that immune reconstitution and reversal of immunosenescence continue longer after ART initiation.^15^

Despite the overall beneficial effect of ART on BTL, concerns remain about regimens containing NRTIs. It has been demonstrated that tenofovir (tenofovir disoproxil fumarate/tenofovir alafenamide fumarate) at therapeutic concentration and abacavir, i.e. two of the most widely used NRTIs, are potent inhibitors of the human telomerase *in vitro*. It has been assumed that this is due to the homology between HIV reverse transcriptase and human telomerase reverse transcriptase, i.e. the enzymatic complex responsible for telomere length maintenance in active proliferative cells.^13–17^ In cross sectional studies of ART-treated PLWH, longer exposure to tenofovir was associated with the inhibition of telomerase activity and significantly shorter BTL *in vitro* in activated PBMCs and *ex vivo* in PBMCs.^18^

Furthermore, longitudinal studies that included long-term virologically suppressed PLWH showed that treatment with tenofovir or abacavir was associated with a smaller gain in whole blood TL than NRTI-sparing regimens.^15^ Consistently, in another study long-term virologically suppressed PLWH treated with tenofovir for at least 4 years had shorter TL in CD8 cells but not in CD4 and had lower telomerase activity in both T cells.^19^

In this study we aimed to investigate whether treatment simplification to a dual therapy with dolutegravir plus lamivudine as the only NRTI might have an impact on BTL after 1 year when compared with a standard triple therapy with an anchor drug plus two NRTIs, one of which was tenofovir disoproxil fumarate/tenofovir alafenamide fumarate or abacavir.

## Material and methods

### Participants

This was a single-site, longitudinal, prospective, matched, controlled study that was carried out from 2018 to 2021. Specifically, we enrolled PLWH who were attending our healthcare facilities at the Department of Infectious Diseases of the University Hospital ‘Fondazione Policlinico Universitario A. Gemelli IRCCS’ in Rome and who met the following inclusion criteria: adults ≥18 years of age who were on stable (i.e. defined as no changes in regimen for ≥12 months) triple standard therapy with a two-NRTI backbone plus an anchor drug and with stable (for ≥12 months) HIV-RNA <50 copies/mL. The study group (dual-therapy, DT) included PLWH who switched to dual therapy with dolutegravir 50 mg plus lamivudine 300 mg once a day based on the clinician’s decision. Participants in the control group (triple therapy, TT) were 1:1 matched with those in the DT group for age, sex and years since HIV diagnosis, time on ART and anchor drug in the triple regimen. PLWH with cancer or currently undergoing chemotherapy were excluded.

Participants were followed from the baseline (BL), which was defined as the date of the treatment switch for the DT group and the date of enrolment for the TT group, to 48 weeks (W48). Demographic, clinical and laboratory variables and therapeutic information were collected from electronic medical records.

### Ethics

The study was approved by our local Ethics Committee (ID 1086). It conformed to the principles laid out in the Declaration of Helsinki and was in accordance with good clinical practice. Written informed consent was obtained from all participants.

### TL assessment

Blood samples were collected in EDTA tubes at both BL and W48. Genomic DNA was isolated from whole-blood specimens using the High Pure PCR Template Preparation Kit (Roche Diagnostics GmbH, Mannheim, Germany) according to the manufacturer’s instructions. BLT was assessed by a monochrome multiplex quantitative PCR assay (MMqPCR) and calculated as the telomere to albumin single copy gene ratio (T/S).^20^ Briefly, the BTL measurements were performed using the CFX96 Touch Real-Time PCR Detection System (Bio-Rad, Hercules, CA, USA). A single source of reference DNA, isolated from the HEK293 cell line, was employed to build the standard curve and used for all the experiments in order to lower interassay variability. The DNA was aliquoted and stored at −80°C until BTL analysis. Each aliquot was used three times to avoid repeated freeze-thaw cycles. The five-point standard curve was performed by 3-fold serial dilution and was included in triplicate in each run together with a reference sample and a negative control. The DNA amount ranged from 150 ng to 1.85 ng per well. At each run we verified both the correlation coefficient (*R*^2^) and the amplification efficiency through the calibration curve. The correlation coefficient was around 0.999 for T and 0.995 for S. The reaction efficiency was calculated from the slope of the standard curves using the equation: E = −1 + 10^(−1/slope)^ and evaluated using software CFX Maestro version 2.0 (Bio-Rad). The efficiency range was 96%–100% for T and 94%–97% for S. If the reaction efficiency fell outside these ranges, the run was rejected. The interassay and intra-assay coefficients of variation (CV%) were 1.07% and 1.02% for T and 1.65% and 0.76% for S, respectively. Each sample was measured twice, each time in triplicate wells. The mean value of the triplicate from each run was averaged. Replicates not meeting the CV% values were discarded, and they were measured a third time; the two closest mean values were averaged and included in the analysis. Samples at the two timepoints belonging to a participant were assessed in the same run.

### Statistical analysis

The participants’ characteristics were described using percentages for categorical variables and median (IQR) for continuous variables. Given the normality distribution of the data, which was evaluated with a Kolmogorov–Smirnov test, BTL was expressed as mean and 95% CIs. The generalized linear model (GLM) was used to evaluate the impact of different treatments on BTL at BL and at follow-up. The GLM tests for independent groups were adjusted for the covariates that were statistically significantly different at BL between the two groups. Collinearity was assessed using variance inflation factors greater than 4.0.

Additionally, a mixed model GLM was used to evaluate repeated measurements of BTL within groups between the two timepoints (i.e. BL and W48).

Multivariable linear regressions were carried out to identify factors associated with BL BTL and factors predictive of BTL changes between BL and W48; variables significantly associated with the BL BTL or BTL change in the univariable analyses were then included in a multivariable model. An alpha level of 0.05 was used to evaluate significance. All statistical analyses were performed in SPSS version 22.0 (IBM, Chicago, IL, USA).

## Results

Overall, 120 subjects were enrolled: 60 in the DT group and 60 in the TT group. The BL characteristics of the participants are summarized in Table 1. Participants’ median age was 53 (IQR 42–59) years and most were men (80%). The median duration of known HIV infection was 13.0 (IQR 5.6–21.9) years, and the median time on ART was 11.8 (IQR 3.8–20.5) years, with a median duration of suppression of 6.9 (IQR 2.8–11.2) years. With regard to ART, emtricitabine/tenofovir was the most common two-NRTI backbone, and integrase inhibitor (INSTI) was the most widely used drug class.

**Table 1.** Baseline characteristics of study participants

	Entire population *n* = 120	DT *n* = 60	TT *n* = 60	*P*
Sex, *n* (%)*				1.000
Male	96 (80.0)	48 (80.0)	48 (80.0)	
Female	24 (20.0)	12 (20.0)	12 (20.0)	
Age, years, median (IQR)*	53 (42–59)	54 (41–59)	53 (42–60)	0.851
Caucasian, *n* (%)	111 (92.5)	56 (93.3)	55 (91.7)	0.729
BMI, kg/m^2^, median (IQR)	24 (21–26)	24 (22–27)	23 (20–26)	0.066
Smokers, *n* (%)^a^	54 (45.0)	28 (46.7)	26 (43.3)	0.714
Alcohol use, *n* (%)^b^	50 (41.7)	25 (41.7)	25 (41.7)	1.000
Subtype				0.114
B	53 (44.1)	22 (36.6)	31 (51.6)	
Non-B	12 (10.0)	8 (13.3)	4 (6.66)	
Unknown	55 (45.8)	30 (50)	25 (41.6)	
Risk factor, *n* (%)				0.366
MSM	51 (42.5)	26 (43.3)	25 (41.7)	
Heterosexual	46 (38.3)	24 (40)	22 (36.7)	
PWID	9 (7.5)	2 (3.3)	7 (11.7)	
Unknown/others	14 (11.7)	8 (13.3)	6 (10.0)	
Time since HIV diagnosis, years, median (IQR)*	13.0 (5.6–21.9)	13.0 (6.0–20.7)	12.9 (4.9–23.9)	0.514
Time on ART, years, median (IQR)*	11.8 (3.8–20.5)	11.5 (3.0–20.2)	12.1 (4.1–20.9)	0.828
Time since last detectable viral load (≥50 copies/mL), years, median (IQR)	6.9 (2.8–11.2)	7.8 (2.3–12.4)	6.7 (2.9–10.6)	0.479
Zenith HIV-RNA, log_10_ copies/mL, median (IQR)	4.9 (4.2–5.5)	4.8 (4.3–5.4)	5.2 (4.2–5.5)	0.316
CD4 cell count nadir, cells/mm^3^, median (IQR)	192 (46–337)	232 (93–381)	112 (35- 295)	0.005
HIV-RNA undetectable (0 copies/mL), *n* (%)	95 (79.2)	49 (81.7)	46 (76.7)	0.500
Previous virological failure, *n* (%)	49 (40.8)	23 (38.3)	26 (43.3)	0.577
CD4 cell count, cells/mm^3^, median (IQR)	695 (517–839)	728 (579–875)	672 (479–780)	0.046
CD4/CD8 ratio, median (IQR)	0.88 (0.57–1.19)	0.93 (0.55–1.22)	0.82 (0.58–1.17)	0.657
Past AIDS-defining events, (CDC C), *n* (%)	29 (24.2)	13 (21.7)	16 (26.7)	0.522
HCV co-infection, *n* (%)	12 (10.0)	3 (5.0)	9 (15.0)	0.053
Comorbidities, *n* (%)				
Hypertension	15 (12.5)	10 (16.7)	5 (8.3)	0.168
Cardiac diseases	24 (20.0)	14 (23.3)	10 (16.7)	0.361
History of cancer^c^	8 (6.7)	4 (6.7)	4 (6.7)	1.000
Diabetes	3 (2.5)	1 (1.7)	2 (3.3)	0.559
Previous CMV, *n* (%)	5 (4.2)	1 (1.7)	4 (6.7)	0.171
Backbone, *n* (%)				0.709
FTC/TDF or FTC/TAF^d^	72 (60.0)	35 (58.3)	37 (61.7)	
ABC/3TC	48 (40.0)	25 (41.7)	23 (38.3)	
Anchor drug in the triple therapy, *n* (%)*				1.000
INSTIs	74 (61.7)	37 (61.7)	37 (61.7)	
Dolutegravir	54 (73.0)	27 (73.0)	27 (73.0)	
Elvitegravir	16 (22.0)	8 (22.0)	8 (22.0)	
Raltegravir	4 (5.4)	2 (5.40)	2 (5.40)	
NNRTIs	38 (31.7)	19 (31.7)	19 (31.7)	
Rilpivirine	30 (79.0)	15 (79.0)	15 (79.0)	
Efavirenz	6 (16.0)	3 (16.0)	3 (16.0)	
Nevirapine	2 (5.0)	1 (5.0)	1 (5.0)	
PIs	8 (6.7)	4 (6.7)	4 (6.7)	
Darunavir/r	6 (75.0)	3 (75.0)	3 (75.0)	
Atazanavir/r	2 (25.0)	1 (25.0)	1 (25.0)	

DT, dual therapy; TT, triple therapy; BMI, body mass index; MSM, men who have sex with men; PWID, people who inject drugs; CDC C, Centers for Disease Control and Prevention stage C (AIDS defining condition); HCV, hepatitis C; CMV, cytomegalovirus; FTC, emtricitabine; TDF, tenofovir disoproxil fumarate; TAF, tenofovir alafenamide fumarate; ABC, abacavir; 3TC, lamivudine; NNRTIs, non-nucleoside reverse transcriptase inhibitors; INSTIs, integrase strand transfer inhibitors; PIs, protease inhibitors; r, ritonavir.

Variable used for 1:1 match.

>10 cigarettes/day.

≥2 alcohol units/day.

Previous non-AIDS related tumour.

TAF *n* = 51 (71.0%) and TDF *n* = 21 (29.0%).

There were no statistically significant differences in the BL characteristics across the two treatment arms except for both nadir CD4 and BL CD4 cell counts, which were significantly higher in the DT group. The main reason for switching to dolutegravir + lamivudine was to make a proactive treatment switch (85%); secondary reasons included toxicity and drug interactions (both 5%) followed by cardiovascular diseases and dyslipidaemia (3.3% and 1.7%, respectively).

At BL, the BTL means were comparable between the two groups: 1.03 (95% CI 0.98–1.08) for DT and 1.02 (95% CI 0.96–1.07) for TT (*P* = 0.973). When we split the population according to the backbone, i.e. emtricitabine/tenofovir disoproxil fumarate-tenofovir alafenamide fumarate (*n* = 72) and abacavir/lamivudine (*n* = 48), we found no difference in the BL BTL [1.04 (95% CI 0.99–1.09) and 1.01 (95% CI 0.96–1.07) (*P* = 0.574)], as well as analysing BL BTL according to tenofovir disoproxil fumarate (*n* = 21) and tenofovir alafenamide fumarate (*n* = 51) [1.05 (95% CI 0.96–1.13) and 1.03 (95% CI 0.97–1.09) (*P* = 0.974)].

All patients completed the follow-up. At W48, the two groups had a similar proportion of subjects with undetectable HIV-RNA and no modifications in CD4 cell counts and CD4/CD8 ratio when compared with the BL (all *P* values non-significant) (data not shown). Of note, when we compared the two groups at W48 the TT group reached a CD4 cell count comparable to that of the DT group [661 (95% CI 467–833) in TT versus 731 (95% CI 568–856) (*P* = 0.137)]. Only one participant in the TT group exhibited a single virological blip, with HIV-RNA of 58 copies/mL at W48. However, the participant reached suppression in the subsequent determination. No virological failure occurred during the follow-up. No participant underwent any ART change during the study period.

**Figure 1.**
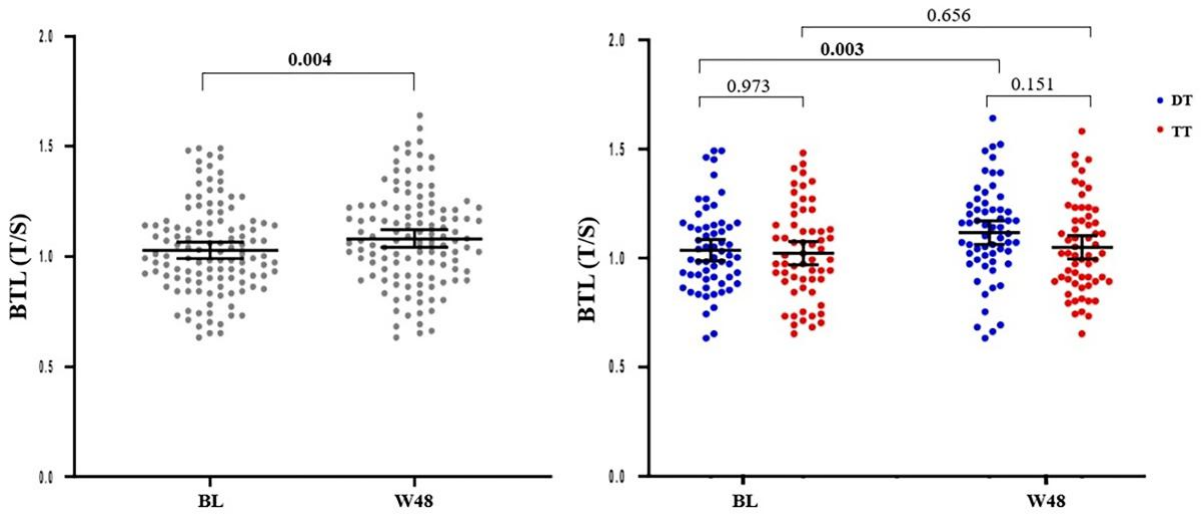
Dynamics of blood telomere length (BTL) expressed as telomere to albumin single copy gene ratio (T/S) at study entry (baseline, BL) and after 1 year (W48) in the entire population (left panel) and in the two groups, dual therapy (DT) and triple therapy (TT) (right panel). Dot plot represents the distribution of BTL. Central horizontal bars represent the mean values and error bars represent the 95% CIs. *P* values between groups at BL and W48 were calculated with an adjusted generalized linear model (GLM), and *P* values intra-group and between the two timepoints with a mixed GLM. Bold denotes statistically significant *P* value. This figure appears in colour in the online version of *JAC* and in black and white in the print version of *JAC*.

Overall, an increase in mean BTL was observed at W48 with respect to BL [+0.161 (95% CI 0.054–0.268) (*P* = 0.004)]. However, a within-group analysis showed a marked mean BTL gain in the DT group [+0.082 (95% CI 0.028–0.135) (*P* = 0.003)], whereas the TT group showed no significant gain [+0.011 (95% CI −0.041 to 0.065) (*P* = 0.656)] (Figure 1).

We also performed a sub-analysis in which we analysed participants who stopped emtricitabine/tenofovir or abacavir separately to determine whether these NRTIs could affect BTL change differently. Participants who stopped abacavir (*n* = 25) showed a significant increase in mean BTL at W48 versus BL [+0.346 (95% CI 0.136–0.556), *P* = 0.003] with respect to participants who stopped emtricitabine/tenofovir (*n* = 35) [+0.174 (95% CI −0.095 to 0.443), *P* = 0.197]; no significant differences in the BTL change between groups were observed (*P* = 0.437) (Figure S1, available as Supplementary data at *JAC* Online). When we further stratified the population according to emtricitabine/tenofovir disoproxil fumarate (*n* = 16) and emtricitabine/alafenamide fumarate (*n* = 19), the BTL change at W48 was not statistically significant (*P* = 0.350 and *P* = 0.954).

In a multivariable regression, being female (versus male, mean change +0.092, 95% CI 0.008–0.176; *P* = 0.032), younger (per 1 year increase, mean change −0.008, 95% CI −0.011 to −0.005; *P* < 0.001) and with a higher CD4/CD8 ratio (per unit, mean change +0.059, 95% CI 0.002–0.118; *P* = 0.052) were independently associated with higher BL BTL (Table S1).

When we explored the predictive determinants of BTL variation between BL and W48, we found that a greater BTL change was associated with the DT group (versus TT mean change +0.059, 95% CI 0.004–0.115; *P* = 0.037), with shorter BL BTL (per unit mean change −0.245, 95% CI −0.391 to −0.109; *P* < 0.001) and with a history of cancer (mean change 0.176, 95% CI 0.060–0.289; *P* = 0.003) (Table 2).

**Table 2.** Determinants of BTL change from baseline to W48 (univariable and multivariable models)

	Univariable			Multivariable		
BTL change	B^a^	95% CI	*P*	B	95% CI	*P*
Group						
TT (ref.)	0					
DT	0.056	−0.006 to 0.117	0.075	0.059	0.004–0.115	0.037
Sex						
Male (ref.)	0					
Female	−0.025	−0.102 to 0.53	0.526			
Age, per 10 years increase	0.019	−0.008 to 0.045	0.166			
Caucasian	−0.087	−0.204 to 0.030	0.143			
BMI	0.004	−0.006 to 0.014	0.443			
Smokers	−0.016	−0.079 to 0.046	0.604			
Alcohol use	0.015	−0.048 to 0.078	0.631			
Subtype						
Non-B	0					
B	−0.044	−0.171 to 0.083	0.492			
Risk factor						
MSM	−0.041	−0.103 to 0.022	0.200			
Heterosexual	0.072	0.012–0.137	0.054			
PWID	−0.031	−0.149 to 0.087	0.600			
Time since HIV diagnosis, per 10 years increase	−0.007	−0.039 to 0.026	0.693			
Time on ART, per 10 years increase	0.001	−0.036 to 0.038	0.958			
Time since last detectable viral load (≥50 copies/mL), per 10 years increase	0.021	−0.038 to 0.079	0.485			
Zenith HIV-RNA, log_10_ copies/mL	−0.016	−0.047 to 0.015	0.307			
CD4 cell count nadir, per 100 cells/mm^3^ increase	0.003	−0.011 to 0.016	0.718			
HIV-RNA						
Detectable (1–49 copies/mL) (ref.)	0					
Undetectable (0 copies/mL)	−0.008	−0.085 to 0.068	0.831			
Previous virological failure	0.010	−0.053 to 0.073	0.750			
CD4 cell count, per 100 cells/mm^3^ increase	−0.003	−0.013 to 0.008	0.584			
Backbone						
TDF/TAF (ref.)	0					
ABC	0.021	−0.043 to 0.084	0.520			
CD4/CD8 ratio	−0.043	−0.100 to 0.013	0.128			
Past AIDS-defining events, (CDC C)	0.014	−0.059 to 0.086	0.707			
BL BTL	−0.297	−0.440 to −0.153	<0.001	−0.245	−0.391 to −0.109	0.001
HCV co-infection, *n* (%)	−0.048	−0.153 to 0.057	0.369			
Comorbidities, *n* (%)						
Hypertension	0.073	−0.020 to 0.166	0.121			
Cardiac diseases	0.028	−0.037 to 0.092	0.399			
History of cancer	0.223	0.106–0.341	<0.001	0.176	0.060–0.289	0.003
Diabetes	−0.109	−0.307 to 0.089	0.279			
Previous CMV	−0.09	−0.245 to 0.064	0.250			

DT, dual therapy; TT, triple therapy; BMI, body mass index; MSM, men who have sex with men; PWID, people who inject drugs; CDC C, Centers for Disease Control and Prevention stage C (AIDS defining condition); HCV, hepatitis C; CMV, cytomegalovirus; TDF, tenofovir disoproxil fumarate; TAF, tenofovir alafenamide fumarate; ABC, abacavir.

^a^Unstandardized coefficient.

## Discussion

In this study, we found that long-term ART-treated virologically suppressed PLWH showed an overall marked gain in mean BTL. However, after 1 year PLWH who simplified the ART to a dual regimen with lamivudine, as the only NRTI, plus dolutegravir had significantly higher BTL gains than participants who continued the standard triple regimen with a two-NRTI backbone (abacavir/lamivudine or emtricitabine/tenofovir disoproxil fumarate-tenofovir alafenamide fumarate).

*In vitro* studies have consistently shown that the NRTIs tenofovir and abacavir inhibit human telomerase, with tenofovir being the most potent inhibitor, and that lamivudine or emtricitabine do not inhibit telomerase at therapeutic concentrations.^17,18^

Moreover, our results are in accordance with data that show that PLWH recipients of long-term ART who maintain virological suppression continue to experience BTL gain many years after having achieved virological suppression.^15^ In a prospective cohort of aviraemic PLWH, Montejano *et al.*^15^ found that after a 2 year follow-up, mean BTL increased significantly in the whole population. However, PLWH treated with a regimen containing tenofovir or abacavir had significantly smaller gains in TL than the participants who were never exposed to tenofovir. In the analysis restricted to participants who were receiving any NRTIs (i.e. mostly treated with abacavir/lamivudine and emtricitabine/tenofovir), tenofovir exposure was not associated with an independent negative effect. Moreover, participants never exposed to tenofovir but treated with two NRTIs also had significantly smaller gains in TL than those receiving NRTI-sparing regimens.^15^

These data are consistent with our results. In fact, at the study entry, the two groups equally exposed to the triple regimen with two NRTIs showed similar BTL. Also, when we compared the BL BTL according to the backbone (abacavir/lamivudine versus emtricitabine/tenofovir disoproxil fumarate-tenofovir alafenamide fumarate) we found no difference, which suggests that both backbones impact BTL equally. However, in the sub-analysis restricted to the group that was simplified we found that the gain was statistically significant in participants who stopped abacavir but not in those who switched from emtricitabine/tenofovir.

In the crude analysis (data not shown) we found that the gain was statistically significant regardless of stopping the backbone, albeit less marked for emtricitabine/tenofovir. It could be hypothesized that the loss of the effect size for tenofovir was due to the reduced sample size in the sub-group.

As expected, BTL was influenced by both age and sex.^21^ In fact, being male and older correlated with shorter BTL, in agreement with the results of a previous study performed with HIV-infected and uninfected individuals.^2^ We found no association with current comorbidities, BMI, smoking habits or alcohol use, all of which can contribute to BTL shortening in PLWH. Previous studies also reported that smoking and metabolic factors had no significant effect on BTL shortening in PLWH.^11^ This might be explained by hypothesizing that these factors could be less of a burden for PLWH or be masked by other factors associated with HIV infection.

Here we also showed that longer BL BTL correlates with a higher CD4/CD8 ratio, which is considered an indirect marker of immunosenescence and a marker of biological age. Regarding the BTL change, PLWH with shorter BL BTL were likely to benefit from a greater gain after 1 year. This might be because in this setting of participants, i.e. with a median of about 12 years of ART and a favourable viro-immunological profile, the magnitude of this phenomenon, i.e. telomere gain, is not as marked at this stage and can be more appreciated in patients with shorter BTL.

Data on suppressed PLWH are controversial. In fact, in a cross-sectional study Montejano *et al*.^22^ reported that there was no association between tenofovir exposure and TL; time of known HIV infection was the only variable associated with shorter TL.

A sub-study of randomized controlled trial patients on darunavir/ritonavir as monotherapy or combined with two NRTIs showed no difference at 144 weeks in terms of telomerase activity and TL between two arms. The authors hypothesized that patients randomized to the monotherapy arm of darunavir/ritonavir could have potentially had greater residual virus replication in sanctuary sites such as the lymph nodes, which may have led to a reduction in TL and therefore would have hidden any favourable effects of cessation of NRTI.^23^

Roy *et al*.^24^ stated that PLWH who use tenofovir alafenamide fumarate show a greater decline in TL than participants who use tenofovir disoproxil fumarate. Differences between the two pharmaceutical forms of tenofovir might occur because tenofovir alafenamide fumarate achieves higher intracellular concentrations than tenofovir disoproxil fumarate. The participants in our study were mostly on tenofovir alafenamide fumarate (51/72 versus 21/72 tenofovir disoproxil fumarate). When we compared BL BTL according to exposure to the two formulations of tenofovir in the baseline ART, we found no significant differences; furthermore, in the sensitivity analysis restricted to the DT group the BTL change was not associated with stopping either tenofovir alafenamide fumarate or tenofovir disoproxil fumarate.

Our study has limitations that warrant consideration. First, it was designed as a longitudinal clinical-based cohort study and as several unmeasured or uncontrolled biases can influence the results of observational non-randomized studies, this should be taken into account when interpreting our results.

Other limitations include the relatively short follow-up and small sample size; the latter could limit the statistical power, especially in some of the smaller sub-group analyses.

However, the research was strengthened by strictly 1:1 matching for several clinically relevant variables, i.e. age, sex, years since HIV diagnosis, time on ART and punctual BL triple regimen (identical composition of the backbone and same anchor drug). These matching variables were selected to potentially minimize the confounders that could impact the BTL variation. This strict 1:1 match allowed us to obtain two fairly homogeneous groups for many variables that could influence the BTL change. Of note, the two groups were also similar for the BTL. However, they differed in nadir CD4 and BL CD4 cell count, and GLM tests adjusted for both variables were performed. As potential confounders, both nadir and current CD4 cell count were explored in the regression analysis; no associations between these variables and BL BTL or changes in the BTL after 48 weeks were observed in this setting.

To minimize the experimental issues, i.e. the intrinsic variability of the BTL measurements, we defined rigorous experimental settings. Moreover, the BTL was normally distributed and with equal variance between the two groups, which allowed us to employ parametric and robust tests.

Furthermore, there was a lack of measurement of TL within each cell subset, which would have allowed us to discover whether the blood TL change is caused by a specific cell type, such as memory and effector CD8 and CD8 T cells. We did not quantify the telomerase activity because the sample source was whole blood and we were unable to isolate PBMCs to extract the total protein.

This study explored the effect on BTL of switching to a dual ART compared with maintaining triple therapy in PLWH. Our analysis showed that in this setting of PLWH with sustained virological suppression, simplification to a dual therapy with dolutegravir plus lamivudine as the only NRTI was associated with a higher gain in BTL than maintaining triple therapy after a 1 year follow-up. As BTL is a well-known indicator of ageing, we believe that these results contribute new data in support of this simplification strategy in terms of its effect on cellular senescence.

## Supplementary Material

dkad237_Supplementary_Data

## Transparency declarations

M.F. received speaker’s honoraria and support for travel to meetings from Bristol-Myers Squibb (BMS), Gilead Sciences, Janssen-Cilag, Merck Sharp & Dohme (MSD) and ViiV Healthcare, and fees for attending advisory boards from BMS, Gilead Sciences and Janssen-Cilag. A.B. received speaker’s honoraria from ViiV Healthcare, and fees for attending advisory boards from Janssen-Cilag. A.C. received support for travel to meetings from ViiV Healthcare. S.D.G. was a paid consultant or member of advisory boards for Gilead Sciences, ViiV Healthcare, Janssen-Cilag, MSD and Bristol-Myers Squibb. All other authors: none to declare.

## Supplementary data

Figure S1 and Table S1 are available as Supplementary data at *JAC* Online.
