# Supplementary material for: Blood telomere length gain in people living with HIV switching to dolutegravir plus lamivudine versus continuing triple regimen: a longitudinal, prospective, matched, controlled study
Source: J Antimicrob Chemother. 2023 Aug 3;78(9):2315–22. doi: 10.1093/jac/dkad237 (PMC10477130; doi:10.1093/jac/dkad237)
Supplement: dkad237_Supplementary_Data [file dkad237_supplementary_data.docx]

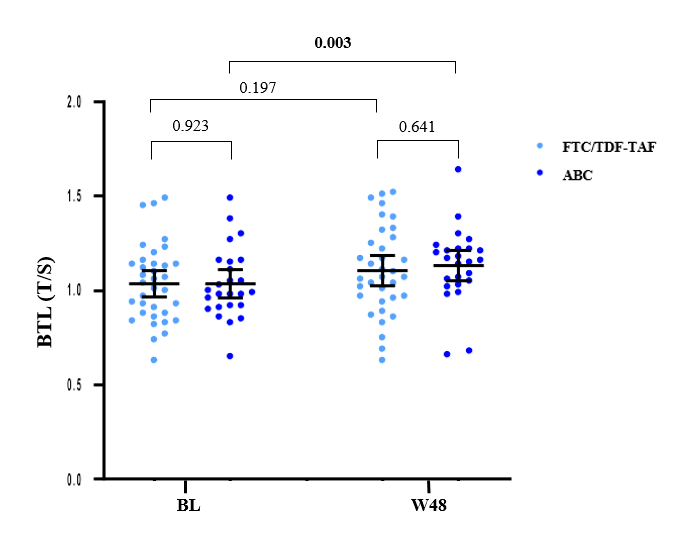


**Figure S1.** Dynamics of blood telomere length (BTL) expressed as telomere to albumin single copy gene ratio (T/S) at study entry (baseline, BL) and after one year (W48) according to the subgroups, i.e., participants who stopped emtricitabine/tenofovir disoproxil fumarate-tenofovir alafenamide fumarate (FTC/TDF-TAF) and abacavir (ABC). Dot plot represents the distribution of BTL. Central horizontal bars represent the mean values and error bars represent the 95% CIs. The p values between groups at BL and W48 were calculated with an adjusted generalized linear model (GLM) and the p values intra group between the two time points with a mixed GLM. Bold value denotes the statistical significance of p.

**Supplementary table S1. Factors associated to baseline BTL (Univariable and multivariable analyses)**

|  | **Univariable** | | | **Multivariable** | | |
| --- | --- | --- | --- | --- | --- | --- |
|  | B | 95% CI | p | B | 95% CI | p |
| **Sex** |  |  |  |  |  |  |
| *Male (ref)* | 0 |  |  |  |  |  |
| *Female* | 0.061 | -0.031/0.152 | 0.193 | 0.092 | 0.008/ 0.176 | 0.032 |
| **Age, per 10 years increase** | -0.079 | -0.107/-0.051 | <0.001 | -0.083 | -0.111/-0.054 | <0.001 |
| **Caucasian** | -0.050 | -0.190/0.090 | 0.48 |  |  |  |
| **BMI** | 0.000 | -0.012/0.011 | 0.953 |  |  |  |
| **Smokers** | -0.019 | -0.094/0.055 | 0.605 |  |  |  |
| **Alcohol use** | -0.002 | -0.077/0.073 | 0.962 |  |  |  |
| **Subtype** |  |  |  |  |  |  |
| *Non-B (ref)* | 0 |  |  |  |  |  |
| *B* | -0.062 | -0.193/0.068 | 0.344 |  |  |  |
| **Risk factor** |  |  |  |  |  |  |
| *MSM* | 0.026 | -0.049/0.100 | 0.492 |  |  |  |
| *Heterosexual* | -0.061 | -0.136/0.014 | 0.11 |  |  |  |
| *PWID* | 0.001 | -0.140/0.140 | 0.997 |  |  |  |
| **Time since HIV diagnosis, per 10 years increase** | -0.027 | -0.066/0.012 | 0.17 |  |  |  |
| **Time on ART, per 10 years increase** | -0.029 | -0.072/0.014 | 0.183 |  |  |  |
| **Time since last detectable viral load (≥50cps/mL), per 10 years increase** | -0.045 | -0.114/0.024 | 0.201 |  |  |  |
| **Zenith HIV-RNA, Log_10_ copies/mL** | -0.002 | -0.038/0.034 | 0.914 |  |  |  |
| **CD4 cell count nadir, per 100 cells/mm^3^ increase** | 0.015 | -0.008/0.038 | 0.185 |  |  |  |
| **HIV-RNA** |  |  |  |  |  |  |
| *Detectable (1-49 cps/mL)(ref)* | 0 |  |  |  |  |  |
| *Undetectable (0 cp/mL)* | 0.038 | -0.4140625 | 0.819 |  |  |  |
| **Previous virological failure** | -0.022 | -0.097/0.053 | 0.567 |  |  |  |
| **CD4 cell count, per 100 cells/mm^3^ increase** | 0.007 | -0.005/0.019 | 0.260 |  |  |  |
| **Backbone** |  |  |  |  |  |  |
| *TDF/TAF (ref)* | 0 |  |  |  |  |  |
| *ABC* | -0.027 | -0.102/0.048 | 0.481 |  |  |  |
| **CD4/CD8 ratio** | 0.083 | 0.018/0.149 | 0.013 | 0.059 | -0.0004/0.118 | 0.052 |
| **Past AIDS-defining events, (CDC C)** | -0.008 | -0.094/0.078 | 0.857 |  |  |  |
| **HCV Co-infection** | -0.008 | -0.134/0.117 | 0.893 |  |  |  |
| **Comorbidities** |  |  |  |  |  |  |
| *Hypertension* | 0.002 | -0.110/0.114 | 0.971 |  |  |  |
| *Cardiac diseases* | -0.022 | -0.098/0.055 | 0.576 |  |  |  |
| *History of cancer* | -0.195 | -0.339/-0.051 | 0.008 | -0.090 | -0.220/0.040 | 0.174 |
| *Diabetes* | -0.003 | -0.239/0.233 | 0.98 |  |  |  |
| **Previous CMV** | 0.180 | -0.167/0.203 | 0.846 |  |  |  |

Abbreviations: BMI, body mass index; MSM, men who have sex with men; PWID, people who inject drugs; HCV, hepatitis C; CMV, cytomegalovirus; TDF, tenofovir alafenamide fumarate; TAF, tenofovir alafenamide fumarate; ABC, abacavir.
